# Integrating Transcriptomics and Metabolomics to Elucidate the Molecular Mechanisms Underlying Beef Quality Variations

**DOI:** 10.3390/foods15030561

**Published:** 2026-02-05

**Authors:** Fengying Ma, Le Zhou, Yanchun Bao, Lili Guo, Jiaxin Sun, Shuai Li, Lin Zhu, Risu Na, Caixia Shi, Mingjuan Gu, Wenguang Zhang

**Affiliations:** 1 College of Animal Science, Inner Mongolia Agricultural University, Hohhot 010018, China; fengyingma1997@163.com (F.M.); zxcvbnm8880314@163.com (L.Z.); byc@emails.imau.edu.cn (Y.B.); sjx9301@163.com (J.S.); lishuai@emails.imau.edu.cn (S.L.); zhulinynacxhs@163.com (L.Z.); narsanjargal@imau.edu.cn (R.N.); caixiashi@imau.edu.cn (C.S.); 2 Key Laboratory of Animal Genetics, Breeding and Reproduction of the Inner Mongolia Autonomous Region, College of Animal Science, Inner Mongolia Agricultural University, Hohhot 010018, China; 3 College of Life Science, Inner Mongolia Agricultural University, Hohhot 010018, China; 13474912747@163.com

**Keywords:** bovine meat quality, HE staining, muscle fiber, transcriptome profiling, metabolome profiling

## Abstract

Elucidating the molecular mechanisms underlying beef quality differences is crucial for precision breeding of high-quality cattle. In this study, we first characterized the myofibrillar morphology of high-quality (H group) and low-quality (L group) beef samples using hematoxylin–eosin (HE) staining. Transcriptomic and metabolomic analyses were then conducted to reveal the molecular regulatory basis of quality variation. HE staining revealed highly significant differences in muscle fiber area and diameter between H and L groups (*p* < 0.01), along with significant differences in muscle fiber density (*p* < 0.05), but no significant differences in muscle fiber perimeter. Furthermore, by focusing on five core metabolic pathways shared across the transcriptome and metabolome datasets, 30 differentially expressed genes (DEGs) and 14 differentially accumulated metabolites (DAMs) were identified. Pearson correlation analysis revealed synergistic regulation between DEGs and DAMs: *AMPD2* modulates umami flavor by regulating inosine accumulation via the purine metabolism pathway; *ACOX3* promotes unsaturated fatty acid synthesis and intramuscular fat deposition through carbohydrate metabolism; genes in the glycolysis/gluconeogenesis pathway maintain post-slaughter muscle pH homeostasis, thereby influencing beef tenderness. Collectively, this study integrates morphological and molecular evidence to elucidate the multi-level basis of beef quality formation, providing key candidate genes, metabolites, and pathways for molecular breeding. These findings offer comprehensive theoretical and technical support for the sustainable development of the premium beef industry.

## 1. Introduction

Beef is a vital dietary source of high-quality protein, vitamins, and minerals, prized by consumers for its distinct flavor and texture [1]. The global beef industry constitutes a major economic sector, driven increasingly by the demand for premium products. As living standards and health awareness rise, consumers seek consistent, superior eating experiences, intensifying the need for precise quality evaluation [2]. Assessing beef quality is multifactorial, encompassing both sensory attributes and physicochemical indicators such as color, marbling, tenderness, juiciness, flavor, and nutritional value [3]. Among these, tenderness is the most important attribute for consumers, as it directly impacts texture and eating experience [4]. Additionally, the aroma and flavor of beef are also important attributes, determined by the composition and concentration of volatile compounds within the muscle tissue [5]. Juiciness is closely related to water-holding capacity and affects chewing texture [6]. In recent years, marbling and intramuscular fat content have emerged as key characteristics of interest to researchers, with intramuscular fat deposition proven to play a crucial role in enhancing meat quality and flavor [7]. However, the formation of beef quality is regulated by an intricate interplay of intrinsic and extrinsic factors, including genetic background, feeding management, nutritional status, and environmental conditions. Despite advances in management practices, the precise molecular mechanisms orchestrating these factors—especially the synergistic regulatory networks underlying muscle fiber characteristics, flavor formation, and tenderness development, as well as their links to gene expression and metabolite accumulation—remain incompletely elucidated [8].

In muscle biology, the biochemical state of skeletal muscle at slaughter and its subsequent metabolic trajectory are the primary determinants of edible quality [9], as skeletal muscle is not merely a structural tissue but a highly active metabolic and endocrine organ. It secretes various bioactive molecules, including specific acylcarnitines, myokines (such as IL-6), and growth factors (such as IGF-1) [10,11]. These can act via autocrine, paracrine, or endocrine pathways to communicate with and modulate the metabolism of adjacent or distant tissues like adipose tissue and liver, potentially pre-conditioning the metabolic state of the animal and thereby influencing the post-mortem transformation of muscle to meat [12,13]. Even under standardized rearing conditions, substantial individual variation in meat quality persists, underscoring the complexity of the underlying molecular regulatory networks. Transcriptomic studies have identified suites of genes associated with key processes such as muscle fiber type specification (shifting between oxidative and glycolytic fibers), lipid droplet biogenesis and turnover, and extracellular matrix remodeling, all of which are correlated with final meat quality outcomes [14,15]. However, gene expression profiles represent a blueprint of potential; they do not guarantee corresponding functional metabolic outputs. The translation of transcriptional signals into phenotypic reality involves multiple post-transcriptional, translational, and metabolic regulatory layers. The metabolome, as the terminal downstream output of biological processes, provides a direct, integrative readout of cellular physiology [16]. It captures the real-time activities of core pathways such as glycolysis, the tricarboxylic acid (TCA) cycle, and lipid metabolism, which are directly responsible for energy status, pH decline, and the generation of taste compounds post-mortem [17]. In fact, single transcriptomic or metabolomic analyses often fail to fully elucidate the molecular basis of phenotypic variation. While transcriptomics captures the transient state of gene expression, it struggles to directly reflect the actual pathways of downstream metabolic flows. Metabolomics, though capable of presenting biochemical endpoints near the phenotypic level, lacks direct interpretation of the upstream gene expression information regulating the accumulation of these metabolites. Therefore, integrating transcriptomics with metabolomics has become a key strategy for systematically deciphering the mechanisms underlying complex trait formation.

In recent years, with the rapid advancement of high-throughput sequencing and metabolomics technologies, the application of transcriptomics and metabolomics in meat quality research has become increasingly widespread [18]. Building on this, transcriptomics and metabolomics have been widely used to uncover key genes, metabolites, and pathways governing muscle growth, fat deposition, and meat quality formation in livestock and poultry. For instance, transcriptomic analysis in swine identified genes influencing intramuscular fat deposition (e.g., *PPP1R3B*, *PPARGC1A*, *SOCS1*, *EIF4E*, *PRKAR2A*, *PRKAG2*, and *FASN*), while metabolomics analysis revealed regulatory associations with metabolites closely linked to meat flavor (e.g., Thr-Leu, Maltol, D-myo-Inositol-4-phosphate, and Fructose-6-phosphate) [19]. Similarly, in studies of chickens and ducks, integrated transcriptomic and metabolomic analyses successfully identified key molecular markers and metabolic pathways influencing meat quality traits [20,21]. Notably, most foundational multi-omics models for meat quality have been established in monogastrics. In contrast, the unique digestive physiology, longer growth cycles, and distinct fat deposition patterns in ruminants like cattle suggest that direct extrapolation from other species may be insufficient. A dedicated, in-depth multi-omics investigation focused specifically on bovine skeletal muscle and its relationship with IMF is crucial to uncover cattle-specific regulatory mechanisms.

To bridge this knowledge gap, we conducted an integrated transcriptomic and metabolomic analysis of Longissimus thoracis muscle from cattle with divergent meat quality. We hypothesized that this approach would reveal coordinated gene–metabolite networks related to meat quality traits, such as flavor, tenderness, and energy metabolism, that are differentially regulated between high- and low-quality groups. Beyond identifying molecular differences, this study aimed to pinpoint key candidate biomarkers and regulatory hubs within these networks. The findings are expected to offer novel insights into the molecular basis of beef eating quality and provide a foundation for developing precision breeding and nutritional strategies to enhance beef quality in a sustainable manner.

## 2. Materials and Methods

### 2.1. Ethics Statement

All experimental procedures in this study complied with the National Research Council’s Guidelines for the Care and Use of Laboratory Animals. All protocols were approved by the Institutional Animal Care and Use Committee of Inner Mongolia Agricultural University (Approval No.: NND2024143).

### 2.2. Experimental Materials and Protocol

The experimental animals were sourced from Inner Mongolia Xuyi Agriculture and Animal Husbandry Co., Ltd. (Bayannur, China). All experimental cattle were fed the same diet and maintained under identical rearing conditions. We selected 14 healthy 3-year-old male beef cattle of similar body weight. Based on preliminary assessment of post-slaughter marbling scores, these cattle were randomly assigned to two groups (*n* = 7 per group): a high-quality meat group (H) and a low-quality meat group (L). This randomized block design, with seven biological replicates per group, provided statistical power to detect between-group differences. Muscle samples were collected under standardized conditions. Within 30 min post-slaughter, approximately 50 g of longissimus dorsi muscle tissue was precisely excised from the area between the 10th and 11th ribs of each carcass. A portion of the tissue samples was immediately frozen in liquid nitrogen for subsequent transcriptome and metabolome sequencing. Another portion of the samples was fixed in 4% paraformaldehyde for slide preparation.

### 2.3. Hematoxylin–Eosin (HE) Staining

HE staining was performed according to the method previously described by Hai et al. [22]. Muscle samples fixed in 4% formaldehyde were cut into 0.5 cm × 0.5 cm pieces and embedded in paraffin blocks, which were then sliced into 2–4 micron sections using a Leica SM 2000 R microtome. After dewaxing, the paraffin sections were stained with hematoxylin and eosin (HE). Subsequently, the sections were dehydrated with gradually increasing concentrations of ethanol and cleared with xylene, observed under an optical microscope (BX53, Olympus Corporation, Tokyo, Japan). ImageJ software (v1.54) was used to quantify the cross-sectional area, diameter, and perimeter of muscle fibers. Measurements were taken for each longissimus dorsi muscle sample, and the average values were calculated for subsequent statistical analysis.

### 2.4. Transcriptome Sequencing

#### 2.4.1. Total RNA Extraction

Total RNA extraction from skeletal muscle tissue was performed using the RNeasy Kit from Qiagen (Hilden, Germany), strictly adhering to the manufacturer’s standard operating protocol. RNA quality was preliminarily assessed by detecting genomic DNA contamination and degradation via agarose gel electrophoresis. RNA integrity was measured using the Bioanalyzer 2100 system from Agilent Technologies (Santa Clara, CA, USA). To meet the requirements for library preparation and sequencing on the DNBSEQ-T7 platform, RNA samples must meet the following quality control standards: concentration ≥ 50 ng/μL, RNA Integrity Number (RIN) ≥ 7.0, OD260/OD280 ratio ≥ 1.8, and total RNA amount ≥ 1 μg. Qualified samples were used for subsequent experiments.

#### 2.4.2. RNA Quantification and Qualification

RNA integrity was evaluated using the Fragment Analyzer 5400.

#### 2.4.3. Library Development and Quality Assurance

Sequencing library preparation utilized total RNA as the starting material, employing BGI (China)’s DNBSEQ-T7RS High-Throughput Sequencing Reagent Kit V3.0 (T7 SM FCL PE100) according to the manufacturer’s standardized protocol. First, mRNA was purified from total RNA using magnetic beads coated with poly-T oligonucleotides. Subsequently, mRNA fragmentation was achieved through high-temperature treatment with divalent cations in the kit’s dedicated first-strand synthesis buffer (5×). First-strand cDNA was synthesized using the kit’s random hexameric primers and M-MuLV reverse transcriptase (RNase H^+^), followed by second-strand cDNA synthesis with the kit’s DNA polymerase I and RNase H. DNA fragments are converted to blunt ends via exonuclease/polymerase activity, followed by 3′-end adenylation. The resulting ends are then ligated to the kit’s proprietary MGIEasy adapter with a hairpin structure. The kit’s MGIEasy DNA Clean Beads magnetic system is used to enrich 250–300 bp cDNA fragments. Following treatment with the kit’s USER enzyme (included) at 37 °C for 15 min and 95 °C for 5 min, the product is amplified using the kit’s Phusion high-fidelity DNA polymerase and universal PCR primers for 15 min at 37 °C followed by 5 min at 95 °C. PCR amplification is performed using the kit’s Phusion high-fidelity DNA polymerase, universal PCR primers, and index primers. PCR products are purified via the MGIEasy DNA Clean Beads magnetic bead system and subjected to library quality assessment using the Qseq 100 instrument. Throughout the experiment, critical reagent parameters such as enzyme quantities and incubation conditions strictly adhered to the DNBSEQ-T7RS High-Throughput Sequencing Reagent Kit V3.0 (T7 SM FCL PE100) protocol. This resulted in high-quality RNA libraries suitable for the DNBSEQ-T7 sequencing platform.

#### 2.4.4. Sequencing Run

After library quality control, different libraries are pooled according to effective concentration and target read yield requirements, then subjected to T7 sequencing to generate 150 bp paired-end reads.

### 2.5. Transcriptome Data Analysis

First, the raw data underwent quality control filtering using fastp software (v0.22.0) to remove read pairs containing adapters. Additionally, read pairs were discarded if single-end reads contained more than 10% N bases or if low-quality bases (Q ≤ 5) constituted over 50% of the sequence. Subsequently, the quality-controlled clean data were aligned to the reference genome Bos_taurus.ARS-UCD1.2 using HISAT2 (v2.2.1). Finally, gene expression quantification was performed using featureCounts (v2.0.6) based on the alignment results.

Differential expression analysis employed the DESeq2 package (v1.44.0) (integrated within the OmicShare platform toolkit). Raw read counts from the count matrix served as direct input data. Data were normalized via Variance Stabilizing Transformation (VST) and analyzed using a negative binomial model for differential expression testing. Differentially expressed genes (DEGs) were screened using the following criteria: adjusted |Fold Change| ≥ 2 and *p*_value < 0.01. The final DEG list was generated for subsequent functional annotation analysis.

### 2.6. Untargeted Metabolomics

Each group of bovine longest back muscle tissue (*n* = 7) was analyzed for off-target metabolomics. Briefly, 50 mg (±5 mg) of sample was weighed, 500 μL of 80% ice methanol solution was added to a 2.0 mL EP tube with a small amount of steel beads, and the tissue was pulverized using a grinder. The samples were then placed in a −20 °C refrigerator for 30 min to precipitate proteins. The samples were then centrifuged at 20,000 rcf for 15 min, 400 μL of supernatant was transferred to a new EP tube and centrifuged again at 20,000 rcf for 15 min, and the final supernatant was transferred to the injection bottle for UPLC-HRMS analysis. All operations were performed on ice. To monitor the stability of the system, QC samples were prepared by mixing 15 μL of extract from each sample medium. The samples were separated on an ultra-high performance liquid chromatography (UPLC) system (UltiMate 3000 UPLC, Thermo, Waltham, MA, USA) with an ACQUITY UPLC T3 (100 mm × 2.1 mm, 1.8 μm) column. The column temperature was set at 45 °C and the flow rate was 0.3 mL/min. The mobile phases consisted of phase A (5 mmol/L ammonium acetate + 5 mmol/L acetic acid + water) and phase B (acetonitrile). During the collection process, the samples were placed in an autosampler with an injection volume of 2 μL for each sample. To minimize the effect of signal fluctuation of the instrument, the samples were analyzed consecutively in a random order, and QC samples were inserted periodically to evaluate the stability of the system and the reliability of the data. Mass spectrometry analysis was performed using a Q-Exactive high-resolution mass spectrometer (Thermo Fisher Scientific, Waltham, MA, USA), and data were collected in positive and negative ion modes, respectively. The parameters of the ion source were set as follows: auxiliary gas pressure of 10, sheath gas pressure of 45, source temperature of 320 °C, spray voltage of +4000 V for positive ion mode and −3000 V for negative ion mode, data-dependent acquisition (DDA) mode with a primary scanning range of 70–1050 Da at *m*/*z*, a resolution of 70,000 (@ *m*/*z* 200), an AGC target of 3 × 10^6^ with a maximum injection time of 100 ms; the first three ions with intensities exceeding 100,000 were selected from the primary spectrum for the secondary fragmentation scan with a secondary resolution of 17,500 (@ *m*/*z* 200), a maximum injection time of 50 ms, and a dynamic exclusion time of 6 s. The first three ions with intensities exceeding 100,000 were selected for the secondary fragmentation scan. QC samples were collected at intervals of 10 samples throughout the analysis for monitoring and correction of systematic errors.

### 2.7. Metabolomics Data Analysis

Data preprocessing for MS involved peak picking, peak grouping, retention time correction, second peak grouping, and annotation of isotopes and adducts using XCMS software (v4.3). LC-MS raw files were converted to mzXML format and analyzed with XCMS, CAMERA, and metaX tools in R. Ion identification relied on retention time (RT) and *m*/*z* values, with peak intensities recorded in a three-dimensional matrix of peak indices, sample names, and ion intensity data.

The HMDB database was utilized to annotate metabolites by comparing the molecular mass data (*m*/*z*) of samples with database entries. Metabolites were annotated if the mass deviation from the database value was within 10 ppm. The molecular formulas of metabolites were confirmed through isotopic distribution analysis. Additionally, an internal library of metabolite fragment spectra was employed for further validation of metabolite identification.

Statistical analysis was conducted using R (v 4.0.0), normalizing raw protein intensity with the “medium” method. Hierarchical clustering was performed with the pheatmap package. Principal component analysis (PCA) was executed using the metaX package. Partial Least Squares Discriminant Analysis (PLS-DA) analysis was carried out using the R package ropls, calculating variable importance in projection (VIP) values for each variable. Pearson correlation coefficient from the cor package was utilized for correlation analysis. The final metabolites with significant differences were screened based on meeting the criteria of *p* Value 1.2 from *t* test, and VIP from PLS-DA analysis.

### 2.8. Enrichment Analysis

Gene set enrichment analysis (GSEA) for transcriptomic data was conducted using GSEA (v4.1.0) and the MSigDB database to identify significantly different gene sets in specific Kyoto Encyclopedia of Genes and Genomes (KEGG) pathways. The criteria for significant enrichment were set as |normalized enrichment score (NES)| > 1, nominal *p* value (NOM *p*-val) < 0.05, and false discovery rate q value (FDR q-val) < 0.25. For metabolomic data, hypergeometric-based enrichment analysis was performed to map differentially accumulated metabolites (DAMs) to KEGG pathways.

### 2.9. Multi-Omics Integration Analysis

By analyzing the KEGG pathway enrichment results, the pathways co-enriched by both histologies were identified using the Wayne diagram. Pearson’s correlation coefficient was applied to calculate the correlation between DEGs and DAMs, and pairs of correlations with a *p* value < 0.05 were screened for heatmap visualization presentation. The presentation of the results of the above analyses was generated through the data analysis and visualization online platforms https://www.omicshare.com/tools/ (accessed on 25 November 2025) and http://imap.metaboprofile.cloud/ (accessed on 26 November 2025).

## 3. Results

### 3.1. Histological Characteristics of the Longissimus Dorsi Muscle

**Figure 1:**
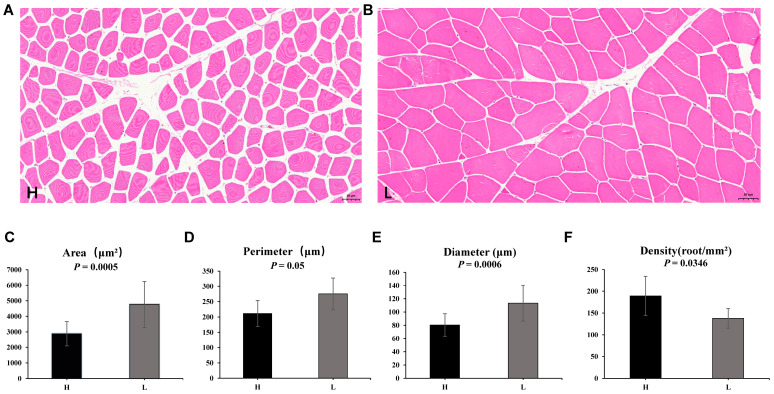
Histological analysis of the longissimus dorsi muscle tissue for two groups with different meat quality levels. (**A**) Muscle fiber characteristics of group H. (**B**) Muscle fiber characteristics of group L. (**C**–**F**) Comparison of muscle fiber area, circumference, diameter, and density between the two groups, *p*-value < 0.01 indicate extremely significant differences group, while *p*-value < 0.05 indicates a significant difference group.

The area, circumference, diameter, and density of muscle fibers with different meat qualities were observed using the HE staining method. The muscle fiber areas of Group H and Group L were 2877.9 μm^2^ and 4766.5 μm^2^, respectively, and the muscle fiber diameters were 80.4 μm and 113.4 μm, respectively. The analysis revealed extremely significant differences in muscle fiber area and diameter indicators between Group H and Group L (*p*-value < 0.01), as well as significant differences in density between the two groups (*p*-value < 0.05). However, there was no significant difference in muscle fiber diameter between the two groups (Figure 1A–F, Table S1).

### 3.2. RNA Sequencing and Identification of Differential Genes

**Figure 2:**
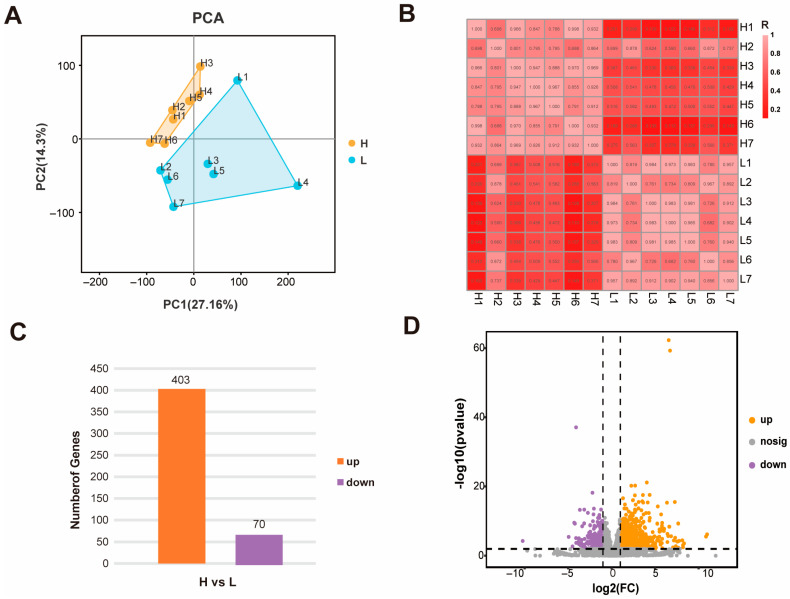
Transcriptome analysis of H vs. L comparison. (**A**) PCA clustering based on gene expression profiles. (**B**) Pearson correlation heatmap of inter-sample expression patterns. (**C**) Bar Chart of DEGs in the H vs. L comparison. (**D**) Volcano plot of DEGs in the H vs. L comparison.

To elucidate the transcriptomic basis of meat quality divergence, RNA sequencing was performed on Longissimus thoracis muscle samples from two groups of cattle exhibiting high (H, *n* = 7) and low (L, *n* = 7) intramuscular fat content. High-throughput sequencing generated a total of 94.4 Gb of clean data, with a Q30 score exceeding 95%, indicating excellent base-calling accuracy (Table S2). The reads demonstrated robust alignment to the bovine reference genome, with mapping rates ranging from 96.09% to 97.21% across all individual samples (Table 1; see read counts per sample in Table S2). Unsupervised PCA revealed clear separation between the H and L groups along the primary principal component, while samples within the same group clustered tightly (Figure 2A). This pattern indicates high intra-group reproducibility and pronounced inter-group transcriptomic differences. Consistent with the PCA, a Pearson correlation matrix illustrated strong correlations among biological replicates within each group, further confirming the reliability of the grouping and the quality of the sequencing data (Figure 2B). Comparative analysis identified a total of 473 DEGs between the H and L groups (*p* < 0.01, |logFC| ≥ 2). Among these, 403 genes were up-regulated and 70 were down-regulated in the high-quality meat group relative to the low-quality group (Figure 2C). The overall distribution of these DEGs, highlighting their statistical significance and magnitude of expression change, is visually summarized in the volcano plot (Figure 2D). This set of DEGs constitutes the core transcriptomic signature associated with divergent beef quality phenotypes.

### 3.3. Enrichment Analysis of DEGs

**Figure 3:**
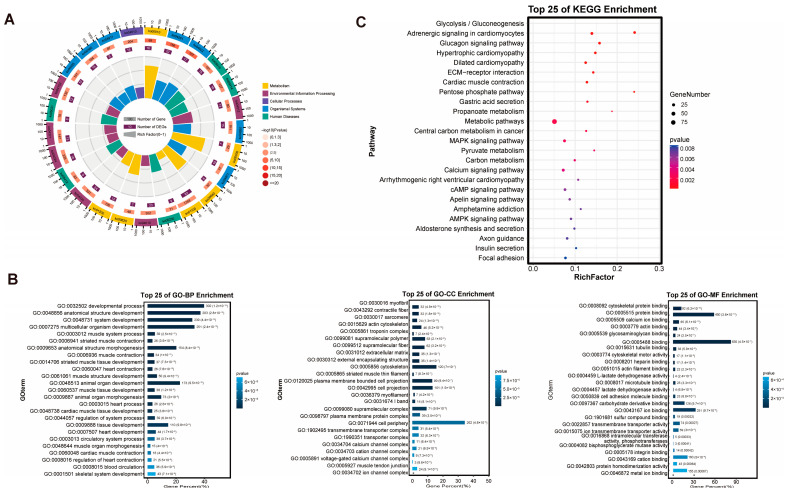
Results of DEGs enrichment analysis. (**A**) GO term enrichment for DEGs in the H vs. L comparison. (**B**) Bar plots show the three independent GO information categories of biological processes, molecular functions, and cellular components, respectively. (**C**) Bubble chart of the top 25 pathway in KEGG analysis of DEGs.

To functionally characterize the 473 DEGs, we performed Gene Ontology (GO) and KEGG enrichment analyses. GO classification revealed that the DEGs were predominantly involved in Biological Process (BP) terms, followed by Cellular Component (CC) and Molecular Function (MF) terms to a lesser extent, as visualized in the circos plot (Figure 3A). Further analysis of the most significantly enriched terms within each category (Top 25) indicated that BP terms were largely related to developmental processes, CC terms were enriched at the cell periphery, and MF terms were primarily associated with binding activities (Figure 3B). KEGG pathway analysis mapped the DEGs to a total of 297 pathways (Table S3). These pathways spanned six major functional categories: Environmental Information Processing (33 pathways, e.g., ECM-receptor interaction, MAPK, Calcium, and cAMP signaling pathways), Organismal Systems (81 pathways), Human Diseases, Cellular Processes (e.g., Focal adhesion, Regulation of actin cytoskeleton), Metabolism (62 pathways, including Glycolysis/Gluconeogenesis, Pentose phosphate pathway), and Genetic Information Processing. Among these, 49 pathways showed significant enrichment (adjusted *p*-value < 0.05). The top 25 most significantly enriched KEGG pathways are presented in Figure 3C. This comprehensive enrichment profile underscores the broad regulatory and metabolic reprogramming associated with divergent meat quality phenotypes.

### 3.4. Metabolomics Analysis and DAMs Identification

**Figure 4:**
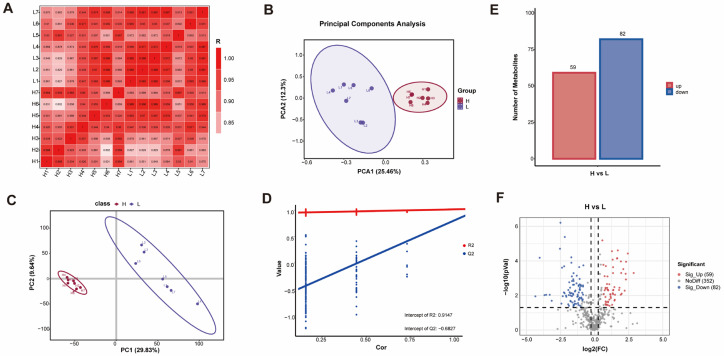
Metabolomics analysis of H vs. L comparison. (**A**) Pearson correlation analysis plot for samples. (**B**) Principal component analysis. (**C**) PLS-DA in different groups. (**D**) PLS-DA model passed the alignment test. (**E**) Bar Chart of Component-Specific Metabolite Quantities. (**F**) Volcano plot of *p*-values between different groups, with red dots representing significantly up-regulated metabolites and blue dots representing significantly down-regulated metabolites.

To delineate the metabolic landscape underlying meat quality divergence, a non-targeted metabolomic profiling was conducted on muscle samples from the H and L groups using LC-MS/MS. Multivariate statistical analyses were first employed to assess data quality and group separation. A sample correlation heatmap demonstrated strong intra-group consistency and clear inter-group distinctions, affirming the reliability of the biological replicates (Figure 4A). Unsupervised PCA revealed a discernible separation trend between the H and L groups along the first principal component (PC1), which explained 25.46% of the total variance, while PC2 accounted for an additional 12.3% (Figure 4B). To maximize the separation based on class membership and identify metabolite drivers of the disparity, a PLS-DA was performed. The PLS-DA score plot showed clear segregation between the two groups (Figure 4C). Validation of the model yielded high parameters (R^2^Y = 0.915, Q^2^ = 0.683), confirming its robustness, goodness-of-fit, and reliable predictive capability (Figure 4D). These results collectively establish a significant biochemical distinction in the muscle metabolome between the two phenotypes. From the total of 493 metabolites detected, differential analysis was performed by applying thresholds of |fold change| ≥ 1.5 and VIP score ≥ 1 from the PLS-DA model. This identified 141 significantly DAMs, comprising 59 upregulated and 82 downregulated metabolites in the H group compared to the L group (Figure 4E,F). The top 10 metabolites ranked by the magnitude of change are listed in Table 2, while the complete lists of all annotated metabolites is provided in Table S4.

### 3.5. Enrichment Analysis of Metabolites

**Figure 5:**
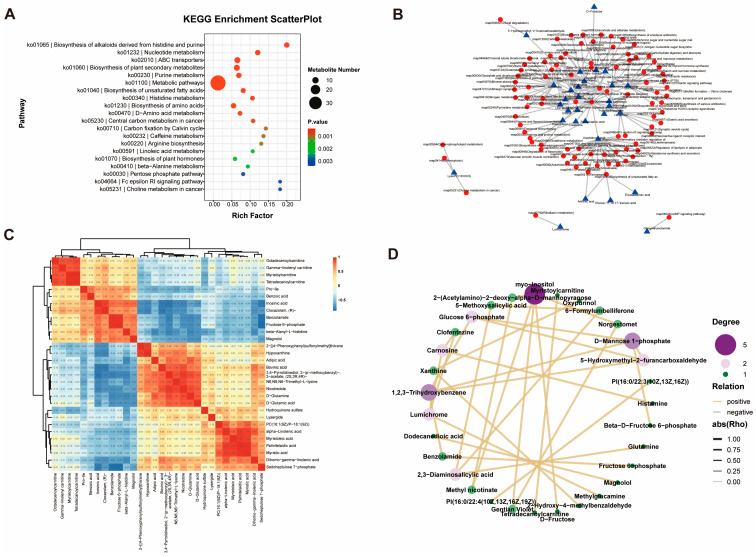
Functional enrichment analysis of differential metabolites. (**A**) Differential metabolite bubble plots. (**B**) Network diagram of regulatory relationships between metabolites and pathways, with triangles representing differential metabolites and dots representing pathways. (**C**) Heat map of significantly different metabolite correlations. (**D**) Correlation network diagram between differential metabolites.

To elucidate the systemic metabolic alterations, the 141 DAMs were subjected to KEGG pathway enrichment analysis. These DAMs were mapped to 119 distinct pathways. Major enriched entries included overarching metabolic pathways, as well as more specific pathways such as Biosynthesis of alkaloids derived from histidine and purine, ABC transporters, and Biosynthesis of plant secondary metabolites (Figure 5A). To visualize the intricate connections between key metabolites and their associated biological pathways, a regulatory network was constructed using the top 30 DAMs ranked by significance. This network (Figure 5B) illustrates how pivotal metabolites—including Pyroglutamic acid, D-Mannose 1-phosphate, Sorbitol, Histidine, Theophylline, Glutamine, and myo-Inositol—participate in and potentially regulate a web of 92 KEGG pathways (e.g., ko00340, ko01110, ko01100). Inter-metabolite relationships were further investigated through correlation analysis. A heatmap of the top 30 most significant DAMs (Figure 5C) revealed distinct clusters of co-regulation. Specific metabolite pairs, such as 6-formylumbelliferone and 5-Methoxysalicylic acid, Oxypurinol and Xanthine, and Clofentezine and Lumichrome, exhibited significant positive correlations (*p* < 0.01). Conversely, a suite of metabolites including 5-Methoxysalicylic acid, Glutamine, 2,3-diaminosalicylic acid, and Theophylline showed significant negative correlations with 7,7,8,8-tetracyanoquinodimethane (*p* < 0.01). Subsequently, a focused association network was generated by filtering for strong pairwise correlations (|r| ≥ 0.7) among the DAMs (Figure 5D). This network highlighted several robust interactions, with the strongest correlations observed between myo-Inositol and Gentian Violet, and between 1,2,3-trihydroxybenzene and both D-Mannose 1-phosphate and 5-Hydroxymethyl-2-furancarboxaldehyde. Synthesizing these multi-faceted analyses, Pyroglutamic acid, Glutamine, Theophylline, myo-Inositol, and D-Mannose 1-phosphate emerge as central, hub-like differential metabolites, likely playing key roles in the metabolic reprogramming associated with meat quality variation.

### 3.6. Combination Analysis of RNA-Seq and Metabolomics

**Figure 6:**
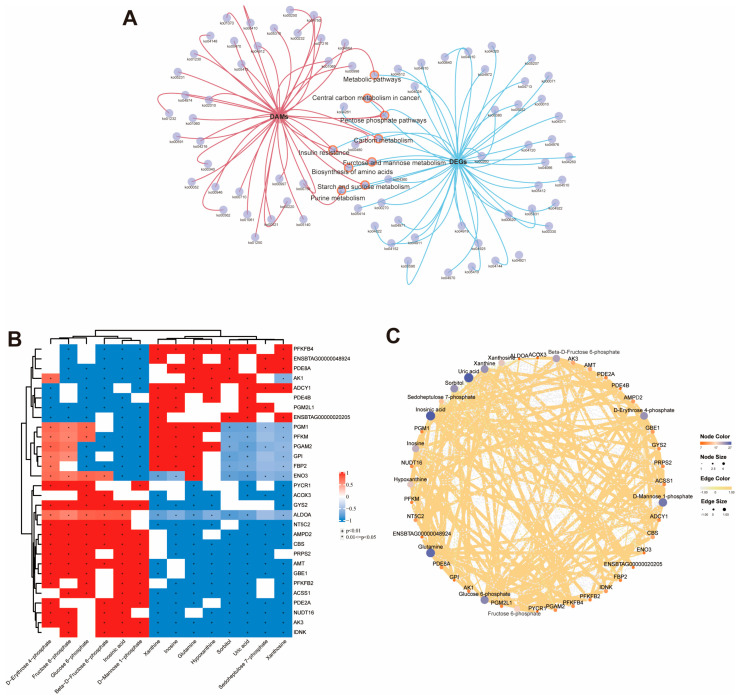
Correlation analysis of DAMs and DEGs related to meat quality. (**A**) Venn diagram for KEGG of DAMs and DEGs. (**B**) Pearson correlation analysis of DEGs and DAMs, Red indicates a positive correlation and blue indicates a negative correlation, + *p* < 0.01, * 0.01 <= *p* < 0.05. (**C**) Correlation Network Diagram of DAMs and DEGs.

To investigate the association between differentially expressed genes and metabolites in skeletal muscle between the H group and the L group, pathway enrichment analysis results were integrated. Forty-nine pathways showed significant enrichment in the transcriptome (*p* < 0.05), and 44 pathways showed significant enrichment in the metabolome (*p* < 0.05). The Venn diagram showed that 9 pathways were shared in the two omics species (Figure 6A, Table 3), among which 5 pathways were related to metabolism, namely: Purine metabolism, Biosynthesis of unsaturated fatty acids, Fructose and mannose metabolism, Carbon metabolism Alanine, Starch and sucrose metabolism. These five metabolism-related pathways involve 14 metabolites and 30 genes. The Pearson correlation method was adopted to demonstrate the correlation between DAMs and DEGs. A correlation coefficient greater than 0 indicates a positive correlation, and less than 0 indicates a negative correlation. The results showed that *PYCR1*, *ACOX3*, *GYS2*, *ALDOA*, *NT5C2*, *AMPD2*, *CBS, PRPS2*, *AMT*, *GBE1*, *PFKFB2*, *ACSS1*, *PDE2A*, *NUDT16*, *AK3*, *IDNK* genes were associated with D-Erythrose 4-phosphate, Fructose 6-phosphate, Glucose 6-phosphate, Beta-D-Fructose 6-phosphate, Inosinic acid, and D-Mannose 1-phosphate was positively correlated (*p* < 0.01), and negatively correlated with other metabolites. On the contrary, *PFKFB*, *ENSBTAG00000048924*, *PDE8A*, *AK1*, *ADCY1*, *PDE4B*, *PGM2L1*, *ENSBTAG00000020205*, *PGM1*, *PFKM*, *PGAM2*, *GPI, FBP2*, *ENO3* genes, and Xanthine and Inosine, Glutamine, Hypoxanthine, Sorbitol, Uric acid, Sedoheptulose 7-phosphate and Xanthosine were positively correlated (*p* < 0.01), and negatively correlated with other metabolites (*p* < 0.01) (Figure 6B,C).

## 4. Discussion

Beef quality, a complex trait determined by multiple factors including muscle fiber characteristics, IMF content, and metabolic profile, is of great economic significance in the livestock industry [8]. Transcriptomic and metabolomic approaches offer powerful means to dissect the molecular basis of phenotypic variation by capturing system-wide changes in gene expression and metabolite profiles [23]. In the present study, we integrated transcriptomic and metabolomic analyses of longissimus dorsi muscle from cattle with high and low meat quality. Our goal was to identify key genes, metabolites, and regulatory pathways. The multi-omics profiling results revealed distinct differences in muscle fiber morphology, gene expression, and metabolic landscapes between the H and L groups. Furthermore, the integrated analysis uncovered intricate molecular networks associated with beef quality divergence.

Muscle fiber characteristics are critical determinants of beef quality, as they directly influence meat tenderness, juiciness, and flavor [24]. The HE staining results demonstrated that the H group had significantly smaller muscle fiber area and diameter, and higher muscle fiber density compared to the L group (*p* < 0.01 or *p* < 0.05). These findings align with numerous previous studies showing that smaller fiber size and higher density correlate with improved meat tenderness [25]. Typically, smaller muscle fibers possess shorter sarcomeres and lower connective tissue content, which reduces the shear force required to chew meat and improves tenderness [26]. The observed differences in muscle fiber characteristics between the H and L groups suggest that muscle fiber development and remodeling may be a key regulatory node in beef quality formation. Transcriptomic analysis further provided molecular evidence for this phenotypic difference, as the DEGs between the two groups were significantly enriched in biological processes related to developmental processes and cellular components at the cell periphery. These DEGs may be involved in regulating muscle cell proliferation, differentiation, and cytoskeletal rearrangement, thereby affecting muscle fiber morphology. For example, genes involved in the regulation of actin cytoskeleton and focal adhesion pathways, which are critical for cell shape maintenance and muscle fiber assembly, were differentially expressed between the H and L groups. This indicates that the transcriptomic reprogramming of muscle fiber development-related genes contributes to the phenotypic differences in muscle fiber characteristics between high and low quality beef.

The functional enrichment of DEGs sheds light on the molecular pathways orchestrating beef quality variation. Pathways including ECM-receptor interaction, MAPK, and calcium signaling are pivotal for muscle growth and homeostasis: MAPK signaling modulates myoblast proliferation and differentiation, while calcium signaling regulates muscle contraction and metabolic adaptation. Notably, the enriched carbohydrate metabolic pathways (e.g., glycolysis/gluconeogenesis, pentose phosphate pathway) underscore the critical role of energy metabolism in shaping meat quality [27]. Our integrated analysis further highlighted that DEGs with strong metabolic are the core regulators of beef quality. For instance, *PGM1* and *PGAM2* were both identified as DEGs and strongly positively correlated with metabolites such as xanthine and hypoxanthine (*p* < 0.01) in our joint analysis. This aligns with a study on Angus cattle, where *PGM1* and *PGAM2* expression levels were correlated with meat color and tenderness, confirming that these genes link the transcriptional regulation of glycolysis and metabolic output, ultimately influencing meat quality [28]. Another key DEG, *ACOX3* showed strong positive correlations with core carbohydrate metabolites, glucose 6-phosphate and β-D-fructose 6-phosphate (*p* < 0.01) in our joint analysis. These two metabolites are pivotal intermediates in glycolysis and glycogen synthesis pathways, linking carbon metabolism to energy supply and biosynthetic processes [29]. This correlation underscores a potential crosstalk between fatty acid metabolism and carbohydrate catabolism: *ACOX3* mediated long-chain fatty acid β-oxidation may coordinate with glucose-derived metabolites to balance energy production and lipid deposition, consistent with a Qinchuan cattle study demonstrating that *ACOX3* regulates IMF accumulation by modulating unsaturated fatty acid synthesis, with high expression associated with higher IMF content and better meat juiciness [30]. The positive association between *ACOX3* and glucose/β-D-fructose 6-phosphate further suggests that enhanced fatty acid metabolism in high-quality beef may be supported by increased carbohydrate flux, ensuring sufficient energy for unsaturated fatty acid biosynthesis and IMF deposition [31].

Metabolomic profiling revealed that the 141 DAMs were significantly enriched in key metabolic pathways, including unsaturated fatty acid biosynthesis and purine metabolism. This aligns with previous multi-omics studies on cattle breeds with divergent IMF content, where metabolites such as specific amino acids and unsaturated fatty acids were identified as key drivers of flavor and nutritional quality [32]. Notably, the DAM inosine acid identified in this study serves as an intermediate in purine metabolism, participating in cellular energy metabolism and signal transduction. Concurrently, inosine acid functions as a key flavor precursor in poultry and livestock meat, engaging in complex interactions with amino acids, sugars, and other substances to generate aromatic compounds that significantly enhance the umami and aroma of meat products. Inosine monophosphate content serves as a crucial indicator for evaluating meat flavor and is a key factor influencing the economic value of poultry and livestock meat products [33,34,35].

The integrated analysis of transcriptomics and metabolomics is crucial for deciphering the complex molecular mechanisms underlying beef quality, linking gene expression changes to metabolite accumulation and effectively establishing gene–metabolite associations. A key finding of this analysis was the identification of 9 common KEGG pathways, among which 5 core metabolism-related pathways (purine metabolism, biosynthesis of unsaturated fatty acids, fructose and mannose metabolism, carbon metabolism, and starch and sucrose metabolism) serve as central regulatory axes governing beef quality traits. Correlation analysis additionally uncovered a well-organized co-regulatory network between DEGs and DAMs, characterized by two distinct yet interconnected modules: one module consisting of anabolic process-related genes, including *ALDOA*, *GYS2*, *PRPS2*, and *AMPD2*, exhibited strong positive correlations with both phospho-sugar intermediates involved in carbon metabolism and glycolysis (e.g., D-erythrose 4-phosphate, fructose 6-phosphate, glucose 6-phosphate) and inosinic acid, collectively driving precursor supply for muscle cell development, metabolism, and umami substance synthesis; the other module, featuring catabolic/inhibitory genes (e.g., *PDE8A*, *PDE4B*, *AK1*), correlated positively with purine degradation products (e.g., xanthine, inosine) and amino acid metabolites such as glutamine. Notably, specific gene–metabolite interactions in these pathways directly or indirectly impact quality traits: in purine metabolism, *AMPD2* is upregulated and catalyzes the conversion of adenosine monophosphate to IMP, a key umami nucleotide, which may promote IMP accumulation to enhance meat flavor [36]; in the biosynthesis of unsaturated fatty acids pathway, differential expression of *ACOX3* may regulate unsaturated fatty acid accumulation, thereby affecting IMF content and fatty acid composition [37]. The global negative correlation between the two modules reflects a coherent metabolic logic: in high-quality beef, cellular resources are preferentially allocated to IMF deposition via coordinated activation of anabolic fluxes and repression of competing catabolic or energy-dissipating processes [38]. Hub molecules within this network, including *PYCR1* (a key regulator of redox metabolism), *PFKFB4* (a critical controller of glycolytic rate), and signaling metabolites such as myo-inositol and glutamine, represent high-priority candidate targets for quality improvement, underscoring the value of integrated multi-omics analysis in unraveling the molecular basis of beef quality.

In conclusion, the integrated transcriptomic and metabolomic analysis revealed distinct differences in muscle fiber characteristics, gene expression, and metabolite accumulation between high and low quality beef. The identified key genes (e.g., *ACOX3*, *ALDOA*, *AMPD2*), metabolites (e.g., inosine, inosine acid, glutamine), and pathways (e.g., purine metabolism, biosynthesis of unsaturated fatty acids, carbon metabolism) provide new insights into the molecular mechanisms underlying beef quality formation. These findings may contribute to the development of molecular markers for beef quality selection and improvement in the livestock industry.

## 5. Conclusions

This study integrated histomorphological and multi-omics analyses to systematically elucidate the mechanisms underlying beef quality differences. HE staining revealed that high-quality beef exhibited significantly smaller muscle fiber area and diameter, as well as higher fiber density, compared with low-quality beef. Transcriptomic and metabolomic analyses further identified five core metabolic pathways as central regulators of beef quality, along with a bipartite co-regulatory network consisting of an anabolic module (*ALDOA*, *GYS2*, *PRPS2*, *AMPD2*) and a catabolic/inhibitory module (*PDE8A*, *PDE4B*, *AK1*). The global negative correlation between these modules reflects a core metabolic logic where cellular resource allocation is optimized to enhance beef quality. Key regulatory targets included *AMPD2* (promoting inosinic acid accumulation for umami), *PFKFB4* (regulating glycolytic rate), and *PYCR1* (mediating redox metabolism), as well as signaling metabolites (inositol, glutamine). Collectively, our findings link muscle fiber morphology to molecular regulatory networks, deepening the understanding of the “gene–metabolite–pathway” axis governing beef quality and highlighting the critical role of anabolic-catabolic balance in high-quality beef formation. The identified core genes, metabolites, and morphological traits provide actionable targets for molecular breeding, underscoring the value of integrated multi-scale analyses in unraveling complex meat quality mechanisms and supporting precision improvement of beef production. In future research, it is expected that a larger sample size and the management of in vitro and in vivo models will functionally validate the prioritized hub genes and metabolites to establish causality.

## Figures and Tables

**Table 1:** Summary of transcriptome sequencing data.

Sample	Raw_Reads	Clean_Reads	Raw_Data (G)	Clean_Data (G)	Mapped Reads	Clean_Q20 (%)	Clean_Q30 (%)	Clean_GC_Content (%)
H1	24,865,257	24,865,257	7.5	7.4	23,950,215 (96.32%)	98.5	95.94	49.62
H2	24,010,082	24,010,082	7.2	7.1	23,157,724 (96.45%)	98.56	96.05	50.03
H3	25,764,292	25,764,290	7.7	7.7	24,767,212 (96.13%)	98.63	96.26	49.68
H4	22,907,080	22,907,080	6.9	6.8	22,226,739 (97.03%)	98.57	96.08	49.41
H5	24,216,215	24,212,017	7.3	7.2	23,459,023 (96.89%)	98.98	95.93	49.89
H6	19,997,243	19,997,243	6	5.9	19,215,351 (96.09)	98.69	96.32	49.82
H7	21,070,085	21,070,085	6.3	6.2	20,347,381 (96.57%)	98.47	96.03	49.14
L1	26,920,506	26,920,506	8.1	8	26,169,424 (97.21%)	98.64	96.26	50.12
L2	19,876,525	19,872,491	6	5.9	19,184,903 (96.54%)	98.95	95.79	49.81
L3	21,215,585	21,215,585	6.4	6.3	20,617,305 (97.18%)	99.14	96.65	50.14
L4	21,647,486	21,644,193	6.5	6.4	20,994,867 (97.00%)	98.95	95.78	49.48
L5	21,761,745	21,757,828	6.5	6.5	21,046,347 (96.73%)	98.97	95.84	49.35
L6	21,322,754	21,322,754	6.4	6.3	20,610,574 (96.66%)	98.53	96.15	48.02
L7	22,531,645	22,531,645	6.8	6.7	21,727,265 (96.43%)	98.69	96.47	49.28

**Table 2:** The top ten differential metabolites in the H vs. L group comparison.

Metabolite	KEGG ID	Log2 FC	*p*-Value	VIP
Berberrubine	NA	7.29	0.00	2.33
5-Hydroxymethyl tolterodine	NA	2.39	0.00	2.06
1,1′-Ethylidenebistryptophan	NA	4.94	0.00	1.99
Brucine	C09084	4.91	0.00	1.92
Norelgestromin	NA	4.85	0.00	2.03
Methylglucamine	NA	4.29	0.00	1.93
Sulfamerazine	NA	3.86	0.00	1.77
Osthol	C09280	3.80	0.00	1.76
Pro-Lys-Ile	NA	3.53	0.00	1.61
Atractylenolide III	C17887	3.47	0.00	1.91

NA indicates no corresponding KEGG ID.

**Table 3:** Shared KEGG pathway between the transcriptome and metabolome.

KEGG Pathway	KEGG ID
Purine metabolism	00230
Metabolic pathways	01100
Biosynthesis of unsaturated fatty acids	01040
Pentose phosphate pathway	00030
Central carbon metabolism in cancer	05230
Fructose and mannose metabolism	00051
Carbon metabolism	01200
Starch and sucrose metabolism	00500
Insulin resistance	04931

## Data Availability

The data presented in this study are available on request from the corresponding authors due to the data used in this study are part of another research project.

## Supplementary Materials

The following supporting information can be downloaded at: https://www.mdpi.com/article/10.3390/foods15030561/s1, Table S1: Morphometric statistics of muscle fibers in two sets of sections. Table S2: Summary of transcriptome sequencing data. Table S3: KEGG enrichment results of DEGs. Table S4: Summary of DMAs.

## Abbreviations

DEG	Differentially Expressed Genes
DAM	Differentially Accumulated Metabolites
HE	Hematoxylin and Eosin
TCA	Tricarboxylic Acid
IMF	Intramuscular Fat
PCA	Principal Component Analysis
GO	Gene Ontology
KEGG	Kyoto Encyclopedia of Genes and Genomes
BP	Biological Process
CC	Cellular Component
MF	Molecular Function
VST	Variance Stabilizing Transformation
FDR	False Discovery Rate
PLS-DA	Partial Least Squares Discriminant Analysis
VIP	Variable Importance in Projection
